# Antioxidant Activities of Plumbagin and Its Cu (II) Complex

**DOI:** 10.1155/2011/898726

**Published:** 2011-10-23

**Authors:** Mingxiong Tan, Yancheng Liu, Xujian Luo, Zhenfeng Chen, Hong Liang

**Affiliations:** ^1^Department of Chemistry and Biology, Yulin Normal College, Yulin 537000, China; ^2^The Key Laboratory for the Chemistry and Molecular Engineering of Medicinal Resources of Ministry of Education, School of Chemistry, Chemical Engineering, Guangxi Normal University, Guilin 541004, China

## Abstract

Plumbagin and its Cu (II) complex [Cu (plumbagin)_2_]**·**H_2_O have been synthesized, and their antioxidant activities towards the inhibitory effect on DPPH free radical, reducing power, total antioxidant capacity, and inhibition on lipid peroxidation were investigated. Plumbagin and its Cu (II) complex were found to exhibit scavenging activities on DPPH radical with the inhibitory rate of 41% and 24%, respectively. The reducing power of plumbagin was outstanding at the concentrations of 1.0, 1.5, and 2.0 mg/mL, compared to Cu (II) complex and synthetic antioxidant 2,6-di-ter-butyl-4-methylphenol (BHT); the highest level reached 1.333 for plumbagin and 0.581 for Cu (II) complex. Also, the inhibition on lipid peroxidation of plumbagin was higher than that of Cu (II) complex and BHT, 46.4% for plumbagin and 24.5% for Cu (II) complex. The results give a strong impact for designing anticancer drugs, combined with their potential cytotoxic and antioxidant activities, which can be targeted selectively against cancer cells and increase their therapeutic index and additional advantages over other anticancer drugs.

## 1. Introduction

Recently, a large number of metal complexes have been designed and tested for anticancer activity and for supportive therapy in cancer patients, including the anticancer drugs doxorubicin, mitoxantrone, bleomycin, and hydroxyurea [1–3]. There is a major debate at present that natural products with diverse bioactivities are becoming an important source of potential (pro)drug chelators [4, 5]. Many naturally occurring molecules such as flavonoids, phenols, and quinones have metal chelating properties, cytotoxic, and antioxidant properties, including the inhibitory effect on free radicals and other damaging oxygen activated products such as the hydroxyl radical, superoxide, hydrogen peroxide, and lipid peroxides [6, 7]. Thus, the prospect of designing chelating prodrugs can be targeted selectively and activated against cancer cells combined with their potential cytotoxic and antioxidant activities increasing their therapeutic index and provides additional advantages over other anticancer drugs [8–10].

**Scheme 1:**
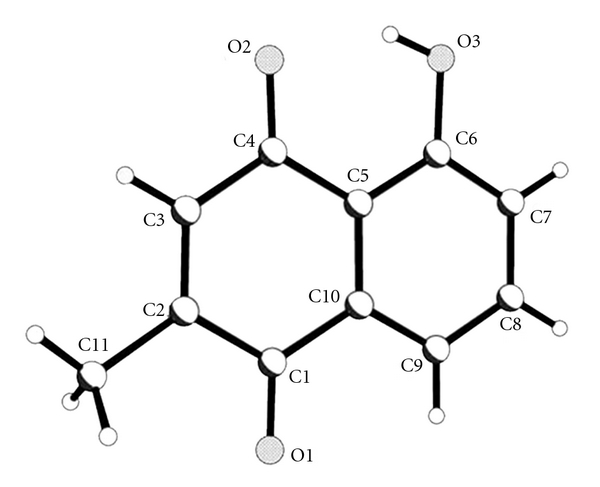
Crystal structure of plumbagin.

**Scheme 2:**
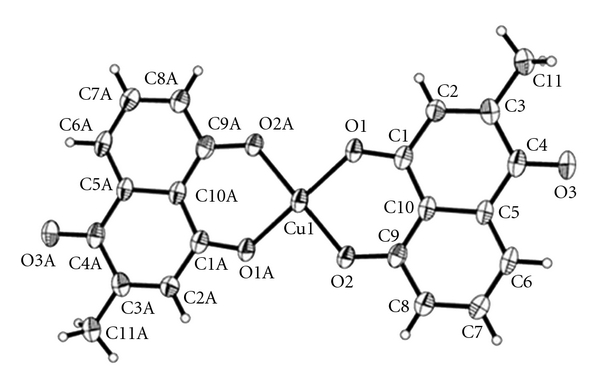
Crystal structure of plumbagin Cu (II) complex.

Our earlier studies have reported the synthesis and anticancer activities of plumbagin (Scheme 1) and its Cu (II) complex (Scheme 2). Plumbagin structurally derived from naphthoquinone was extracted from *Plumbago zeylanica L,* a Chinese traditional medicine. It was found that both plumbagin and its Cu (II) complex exhibited significant antitumor activities against seven human tumour cell lines (BEL-7404, NCI-H460, CNE-2, 786-O, MCF-7, HCT-116, and Hep-G2), and Cu (II) complex possessed significantly enhanced antitumor with respect to the free ligand Cu (II) ion plays an important role [11]. All of these results make it interesting to study the antioxidant activities of plumbagin and its Cu (II) complex.

## 2. Materials and Methods

UV-Vis absorption spectra were performed on a Varian Cary100 UV-Visible spectrophotometer. 2,2′-Diphenyl-1-picrylhydrazyl (DPPH) was purchased from Sigma Chemicals Co. All the metallic salts were purchased from Alfa Co. Ltd. The solvents were analytical grade. All the materials were used as received without further purification unless noted specifically.

Plant material, roots of *P. zeylanica*, was collected in Guangxi province and identified by Prof. Tang (Institute of Life Science, Guangxi Normal University).

### 2.1. Isolation and Structure Identification of PLN

The roots of *P. zeylanica *(15 kg) were extracted with 95% EtOH. The solvent was concentrated in vacuo, and the residue was successively partitioned between H_2_O and n-hexane followed by EtOAc. The EtOAc extract (252 g) was subjected to silica gel cc with a gradient of EtOAc in n-hexane, eluting with n-hexane-EtOAc (9 : 1) to yield an orange crystal, plumbagin (1.33 g).

### 2.2. Synthesis of [Cu(Plumbagin)_2_]·H_2_O

This yellowish brown of plumbagin Cu (II) complex was synthesized by the same method as the reference described by Chen et al. [11].

### 2.3. Scavenging Activity on DPPH Radical

To evaluate the free radical scavenging activity, plumbagin or Cu (II) complex solution was allowed to react with a stable free radical, 2,2′-diphenyl-1-picrylhydrazyl radical (DPPH) [12]. 1 mg/mL of plumbagin or Cu (II) complex in different reaction times (10, 20, 30, 40, 50, and 60 min) was assayed, and 1 mg/mL of synthetic antioxidant 2,6-di-ter-butyl-4-methylphenol (BHT) used for comparison. The reaction mixture was incubated at 25°C. The scavenging activity on DPPH radical was determined by measuring the absorbance at 515 nm each 10 min until the reaction reached the steady state. The antioxidant activity was expressed as a percentage of scavenging activity on DPPH radical: SC% = [1 − (absorbance of sample)/(absorbance of control)] × 100%.

### 2.4. Reducing Power

The determination of reducing power was performed as described by Oyaizu [13]. The reducing power of various plumbagin and Cu (II) complex solutions (0.5, 1.0, 1.5, and 2.0 mg/mL) was investigated; BHT was also determined for comparison. The various samples were mixed with phosphate buffer (2.5 mL, 0.2 M, pH 6.6), potassium ferricyanide (2.5 mL, 1%) was incubated at 25°C for 20 min, trichloroacetic acid was added, and the mixture was centrifuged at 3000 rpm for 10 min. The upper layer of solution (2.5 mL) was mixed with distilled water (2.5 mL) and ferric chloride (0.5 mL, 0.1%), then the absorbance was measured at 695 nm against a blank.

### 2.5. Determination of Total Antioxidant Capacity

The total antioxidant capacity was determined according to the method of Prieto [14]. Plumbagin or Cu (II) complex (0.5 mg/mL) in different reaction times (20, 40, 60, 80, 100, and 120 min) was combined with 0.3 mL of reagent solution (0.6 M sulphuric acid, 28 mM sodium phosphate, and 4 mM ammonium molybdate). The reaction mixture was incubated at 95°C for 150 min. After the mixture had been cooled to room temperature, the absorbance of the mixture was measured at 595 nm against a blank. The readings were taken each 30 min. The antioxidant activity of BHT (0.5 mg/mL) was also assayed for comparison.

### 2.6. Antioxidant Potential of Plumbagin or Cu (II) Complex in Peanut Oil

Calculated amounts of plumbagin or Cu (II) complex solution were added to 50 mL of peanut oil. The additive was mixed into the oil with a magnetic stirrer. The oxidative deterioration of samples was studied using Schaal oven test method as described by Economou et al. [15]. The oil samples (50 mL each) were placed in open 100 mL beakers and placed in 60 ± 0.5°C oven for 24 h. The sample (1 mg/mL) was prepared under the same conditions treated with different reaction times (1, 2, 3, 4, 5, and 6 days), BHT (1 mg/mL) was also assayed for comparison. The rate of antioxidant of peanut oil was estimated according to the increase of 2-thiobarbituric acid-reactive substances (TBARS) using the classical TBA procedure. The TBARS values of untreated and treated samples were used to calculate the inhibition of lipid oxidation as follows: Inhibition (%) = (control-treatment)/control × 100%.

### 2.7. Statistical Analysis

All experimental results were centred at using three parallel measurements of mean ± SD. Analysis of variance was performed by ANOVA procedure. Duncan's new multiple-range test was used to determine the differences of means. *P* values < 0.05 were regarded as significant and *P* values < 0.01 as very significant.

## 3. Results and Discussion

### 3.1. Scavenging Activity on DPPH Radical

Antioxidant properties, especially radical scavenging activities, are very important due to the deleterious role of free radicals in biological system. DPPH is a kind of stable free radical and accepts an electron or hydrogen radical to become a stable diamagnetic molecule [6], which was widely used to investigate radical scavenging activity now for its advantage of ease and economy. In DPPH radical scavenging assay, antioxidants are able to reduce the stable DPPH radical to yellow-coloured, and the antioxidant power is indicated by the degree of discoloration which could be determined by measuring of a decrease in the absorbance at 515 nm.

**Figure 1:**
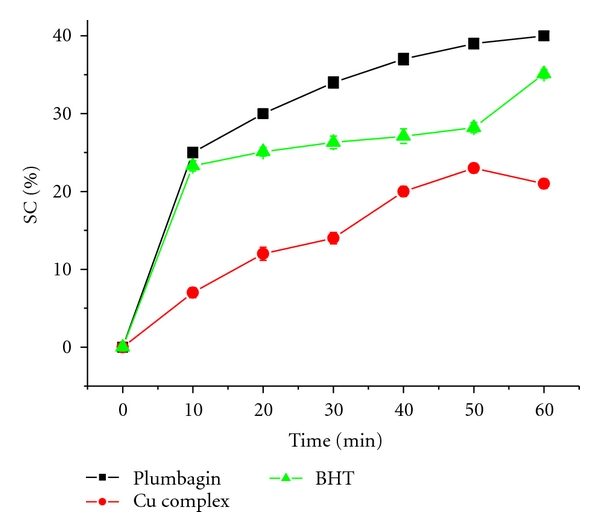
DPPH free radical scavenging activity of plumbagin, Cu (II) complex, and BHT in different reaction times (10, 20, 30, 40, 50, and 60 min).

As Figure 1 shows, at the concentration of 1 mg/mL of plumbagin or Cu (II) complex possessed scavenging activities on DPPH radical with the inhibitory rate of 41% and 24%, respectively, their scavenging effect increased with increasing reaction time, and plumbagin scavenging activities are pretty, more higher than its Cu (II) complex and BHT, indicating that the presence of Cu (II) decreased the scavenging effect of DPPH radical.

### 3.2. Reducing Power

**Figure 2:**
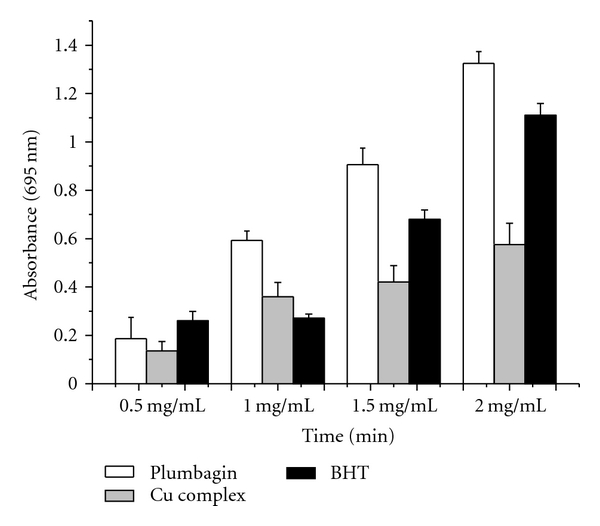
The reducing power of plumbagin, Cu (II) complex and BHT treated with different solution concentrations (0.5, 1.0, 1.5, and 2.0 mg/mL).

The reducing capacity of a compound may serve as a significant indicator of its potential antioxidant activity [16]. In the assay, the presence of reductants in the antioxidant sample causes the reduction of the Fe^3+^/ferricyanide complex to the Fe^2+^/ferrous form [17], so the reducing power of the sample could be monitored by measuring the formation of Perl's Prussian blue at 695 nm [18]. As Figure 2 shows, the reducing properties of plumbagin and Cu (II) complex at different concentration exhibited correlation with increasing concentration. The reducing power of plumbagin was outstanding at the concentration of 1.5 and 2.0 mg/mL compared to Cu (II) complex and BHT. At the concentration of 2.0 mg/mL, the reducing power of plumbagin and Cu (II) complex reached 1.333 and 0.581, respectively. It was speculated that the decreased antioxidant power of Cu (II) complex, compared to plumbagin, may be the absence of phenolic group due to forming the complex, thus the presence of the active hydrogen in phenolic hydroxyl group was likely to contribute considerably to the observed antioxidant effects.

### 3.3. Total Antioxidant Capacity Assay

**Figure 3:**
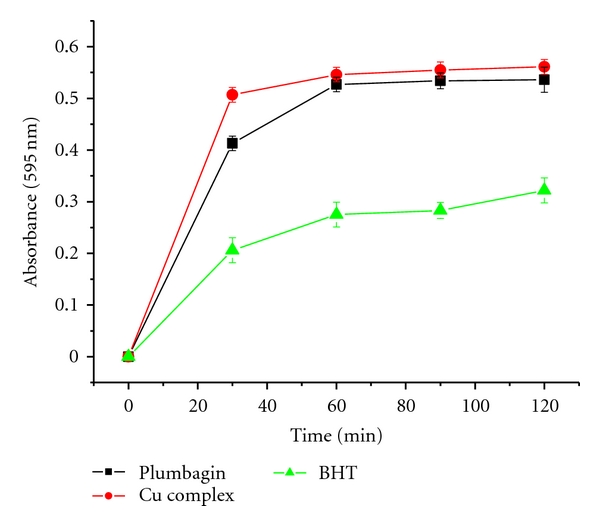
The total antioxidant power of plumbagin, Cu (II) complex, and BHT in different reaction times (20, 40, 60, 80, 100, and 120 min).

The assay is based on the reduction of Mo (VI) to Mo (V) by plumbagin and Cu (II) complex in different reaction times (20, 40, 60, 80, 100, and 120 min) and subsequent formation of a green phosphate/Mo (V) complex at acid pH. The high absorbance values indicated that the sample possessed significant antioxidant activity. As Figure 3 shows, plumbagin and Cu (II) had significant total antioxidant activities compared to BHT, and the effects increased with increasing reaction time, while total antioxidant capacity of Cu (II) complex was superior to plumbagin.

### 3.4. Lipid Peroxidation in Peanut Oil

**Figure 4:**
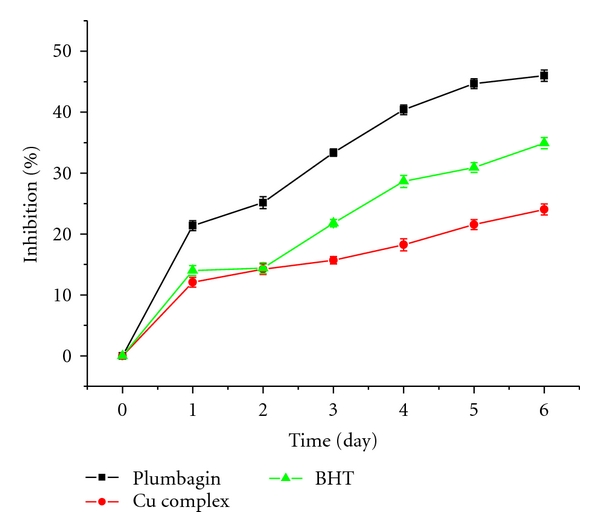
Lipid oxidation inhibition by plumbagin, Cu (II) complex, and BHT in peanut oil in different reaction times (1, 2, 3, 4, 5, and 6 days).

The level of 2-thiobarbituric acid-reactive substances (TBARS), products of lipid peroxidation, is often measured in order to assess the extent of oxidation that occurs in biological systems. Peanut oil was treated with plumbagin, Cu (II), and BHT complex at the concentration of 1 mg/mL in different reaction times (1, 2, 3, 4, 5, and 6 days). As shown in Figure 4, the inhibition rate of lipid oxidation between plumbagin and Cu (II) complex had significant effects and was enhanced with increasing reaction time, the highest level reached 46.4% for plumbagin and 24.5% for Cu (II) complex after 6 days, and the lipid oxidation inhibition of Cu (II) complex is lower than that of PLN and BHT at the same period. 

## 4. Conclusions

In this study, we have investigated the antioxidant possibility of plumbagin and its Cu (II) complex. The results indicated that both the compounds exhibited some antioxidant activities, including the inhibitory effect on DPPH free radical, reducing power, total antioxidant capacity, and lipid peroxidation, which plumbagin possessed higher abilities than that of Cu (II) complex and BHT at the same condition towards DPPH free radical, reducing power, and lipid peroxidation. It was speculated that the decreased antioxidant power of Cu (II) complex compared to plumbagin may be the absence of phenolic group due to forming the complex, thus the presence of the active hydrogen in phenolic hydroxyl group was likely to contribute considerably to the observed antioxidant effects. Also, this study gives a strong impact for designing anticancer drugs, combined with their potential cytotoxic and antioxidant activities, which can be targeted selectively against cancer cells and increase their therapeutic index and additional advantages over other anticancer drugs.
